# Determinants of neonatal hypoglycemia among neonates admitted to neonatal intensive care units of public hospitals in Wolaita Zone, Southern Ethiopia, 2023: An unmatched case–control study

**DOI:** 10.3389/fped.2024.1429066

**Published:** 2024-08-20

**Authors:** Dawit Tesfaye Wodajo, Woiynshet Gebretsadik, Zeleke Aschalew Melketsedik, Nathan Desalegn, Fikre Moga Lencha

**Affiliations:** ^1^ College of Health Sciences and Medicine, Wolaita Sodo University, Sodo, Ethiopia; ^2^ College of Medicine and Health Sciences, Arba Minch University, Arba Minch, Ethiopia

**Keywords:** neonatal, hypoglycemia, determinants, case–control, Wolaita

## Abstract

**Background:**

Globally, neonatal hypoglycemia is a common child health problem and significantly contributes to morbidity and mortality, with its impact being particularly detrimental in developing countries. Although being a prevalent metabolic condition, it is frequently overlooked. Furthermore, the problem is not adequately studied in Ethiopia, as seen by a few published studies on the topic, highlighting the lack of knowledge about its determinants.

**Objective:**

This study aims to assess the determinants of neonatal hypoglycemia among neonates admitted to neonatal intensive care units of public hospitals in Wolaita Zone, Southern Ethiopia, 2023.

**Methods:**

An institution-based unmatched case–control study design was conducted among 249 (83 cases and 166 controls) participants. Data were collected from 29 March to 23 May 2023 using a pretested chart review extraction tool/checklist. A consecutive sampling method was used for participant selection. Data were analyzed using Statistical Package for Social Science version 25. Binary logistic regression analysis was used to identify the determinants of neonatal hypoglycemia, and statistical significance was declared at *P* < 0.05.

**Results:**

A total of 83 cases and 166 controls were included in the study. The following factors were significantly associated with neonatal hypoglycemia: preterm neonates [adjusted odds ratio (AOR) 4.888 (95% confidence interval, CI, 1.113–21.478)], age of the neonate at admission (3–5 h) [AOR 4.205 (95% CI 1.852–9.547)], hypothermia [AOR 5.485 (95% CI 2.360–12.748)], late initiation of breastfeeding [AOR 6.229 (95% CI 2.665–14.599)], mode of delivery [AOR 5.034 (95% CI 1.688–15.011)], and small for gestational age [AOR 3.645 (95% CI 1.286–10.330)].

**Conclusion and recommendation:**

In the current study, numerous determinants of neonatal hypoglycemia have been identified. Therefore, it is crucial to give due emphasis to providing comprehensive health education to mothers regarding effective breastfeeding methods, strategies to avoid neonatal hypoglycemia, and the various factors that heighten its likelihood. In addition, healthcare professionals should emphasize implementing preventive measures to prevent this grave condition.

## Introduction

According to a recent report by the World Health Organization (WHO), approximately 2.4 million children globally lose their lives within the first month after birth. This distressing statistic translates to around 6,700 newborn deaths every day, accounting for 47% of all children under the age of 5. This figure has increased from 40% in 1990, highlighting the urgent need for targeted interventions to address this issue (1).

Approximately 42% of all neonatal deaths worldwide occur in sub-Saharan Africa (SSA) (2). In Ethiopia, the 2019 Ethiopian Mini Demographic Health Survey (EMDHS) indicated that the neonatal mortality rate (NMR) has risen to 30 deaths per 1,000 live births from 29 deaths per 1,000 live births reported in the 2016 Ethiopian Demographic Health Survey (EDHS) (3). These alarming rates of neonatal mortality are deeply connected to preventable factors, including neonatal hypoglycemia (NH), which stands as the most prevalent metabolic disorder among newborns.

On a global scale, the incidence of postpartum hypoglycemia in newborns ranges from 5% to 15%. The prevalence of this condition is increasing due to factors such as preterm births, maternal diabetes, and obesity, all of which pose a risk (4). It is estimated to affect 1–5 out of every 1,000 live births, with higher rates observed in populations deemed to be at higher risk (5, 6).

Neonatal hypoglycemia is frequently observed as a co-existing condition with prematurity, birth asphyxia, intrauterine growth retardation, infants born to diabetic mothers, and sepsis. It presents a significant challenge to the health of children and demands urgent attention. If left unaddressed, the long-term consequences can be devastating. Despite its high prevalence and its substantial impact on infant mortality, it is often undervalued and overlooked (7, 8).

Neonatal hypoglycemia exhibits a higher occurrence in economically disadvantaged countries like Ethiopia, potentially due to the elevated rates of low birth weight (LBW) or intrauterine growth retardation (IUGR) and inadequate feeding practices or suboptimal nursing support (9). Across Africa, the prevalence of neonatal hypoglycemia spans from 2.2% to 30.5% (10–13). In the context of Ethiopia, the prevalence ranges from 21.2% to 25% (8, 14–15).

Based on a study conducted in Ethiopia, neonatal hypoglycemia contributes to approximately 7.7% of all reported fatalities (16). In addition, another study revealed that neonatal hypoglycemia ranks among the top 10 causes of both admission (17.7%) and mortality (11.9%) among newborns (17). This evidence highlights the importance of identifying the variables that influence neonatal hypoglycemia, as this understanding can facilitate early prevention, accurate clinical diagnosis, and targeted therapies aimed at reducing both morbidity and mortality (15, 16).

Neonatal hypoglycemia is widely recognized as a prevalent child health problem and a significant cause of hospital admissions and mortality globally, including in the study area. However, the factors influencing its occurrence have received inadequate attention and limited investigation. Previous studies on the subject have been hindered by the limitations of their cross-sectional design, which do not establish a causal relationship between hypoglycemia and its determinants. These studies were also conducted in a single institution, which may be susceptible to unrepresentativeness. Consequently, previous research has called for a case–control study across multiple centers (14, 15). Notably, this present study represents the first of its kind to examine the determinants of neonatal hypoglycemia in the specific study area and contributes to a broader understanding of this condition in Ethiopia.

## Methods and materials

### Study area and period

The study was carried out in the Wolaita Zone, an administrative Zone in South Nations and Nationalities of People Region (SNNPR), Ethiopia. The zone has 68 health centers, eight public primary hospitals, and the Wolaita Sodo University Comprehensive Specialized Hospital. The eight hospitals are Bedesa, Tabala, Bombe, Halale, Bitena, Gesuba, Bele, and Boditi primary hospitals. The study was conducted at public hospitals providing neonatal intensive care unit (NICU) services in the Wolaita Zone from 29 March to 23 May 2023.

### Study design

An institution-based, unmatched case–control study design was employed.

### Populations

The source population for this study comprised all neonates admitted to the NICUs of public hospitals in Wolaita Zone. The study population included those neonates admitted to the NICUs during the study period who met the inclusion criteria for both cases and controls. The sample population consisted of cases defined as neonates in the NICUs with a random blood sugar (RBS) level of <47 mg/dl and controls defined as those with a random blood sugar level of ≥47 mg/dl. Inclusion criteria required that all neonates admitted to NICUs with random blood sugar levels <47 mg/dl for cases and ≥47 mg/dl for controls had their mothers or primary caregivers present and willing to participate during data collection. Neonates aged ≤2 h and those whose mothers were critically ill were excluded from the study.

### Sample size determination

The sample size was calculated by using the double population proportion formula with the STATCALC application of Epi-info version 7 statistical software. Birth asphyxia, prematurity, and LBW were considered determinants of neonatal hypoglycemia. Among these factors, LBW yielded the maximum sample size, so it was used to determine the final sample size (18). The sample size was calculated by using the following assumptions: proportion of neonates of controls with LBW: 69.91%; proportions of neonates of cases with LBW: 87.3%, odds ratio from the study in Central Java: 2.97 (18); type I error: 5%, power: 80%, case-to-control ratio: 1%:2%; and non-response rate: 10%. Thus, the total sample size was 249, with 83 cases and 166 controls.

### Sampling technique and procedure

The research was carried out in all public hospitals providing NICU services in the Wolaita Zone. However, due to the absence of NICU services in the newly opened primary hospitals (Tabala and Bedesa primary hospitals), they were excluded from this study. Therefore, the study included Wolaita Sodo University Comprehensive Specialized Hospital (WSUCSH), Boditi Primary Hospital, Gesuba Primary Hospital, Bele Primary Hospital, Bombe Primary Hospital, Halale Primary Hospital, and Bitena Primary Hospital. Then, the calculated sample size was proportionally allocated to each hospital based on the average monthly admission of the cases from the previous same month's report. A consecutive sampling method was used to select both cases and controls.

### Data collection instruments and procedures

Seven qualified BSc nurses were recruited to collect the data, and one public health officer was recruited for supervision. Training was provided to the data collectors and the supervisor on data collection techniques, the purpose of the study, and data recording. Data were collected using a pretested chart review extraction tool, and the mothers were interviewed for some sociodemographic variables. The weight for the gestational age percentile was calculated using the Omni calculator. The data collection instrument was prepared by reviewing different related literature works that are sufficient to address the study objectives. The chart review extraction tool consists of four sections: sociodemographic characteristics (8 items), maternal characteristics (5 items), obstetric characteristics (7 items), and neonatal characteristics (14 items).

### Collection of blood samples

In this study, the neonates’ RBS was measured by health works in the study hospitals using a glucometer and a quick glucose test strip. The blood collection method involved the following steps: the neonate’s right or left heel was cleaned with cotton wool and 70% methylated alcohol. A sterile lancet was then used to puncture the heel after the cleaned area had air-dried for 30 s. A glucose test strip attached to a glucometer was utilized to make contact with the pooling blood at the puncture site, and the blood sugar was read in about 10 s. Finally, the healthcare workers recorded the results on the patient's card for documentation. Our data collectors subsequently utilized this documented information from the patient cards to analyze the blood glucose levels of the neonates in our study.

### Operational definition and measurements

#### Cases

Cases were defined as those with random blood sugar concentrations of <47 mg/dl for any postnatal age during the study period (11, 19).

#### Controls

Controls were defined as those with random blood sugar concentrations of ≥47 mg/dl for any postnatal age during the study period.

### Data quality control

To ensure data quality, training was provided to the supervisor and data collectors on the purpose of the study and data collection techniques. A pretest was conducted at a location outside of the study area (Arba Minch General Hospital) on 5% of the total sample size. The required modifications and fixes were done following the results of the pretest. The supervisor and investigator monitored the day-to-day data collection process to ensure the completeness and consistency of collected data. The chart extraction tool was checked for consistency and completeness. Before analysis, data pre-analysis tasks like data cleaning, coding, recording, and variable re-categorization were performed.

### Data processing and analysis

Data were checked for completeness and consistency before data entry; the data were then entered into Epi data 4.6 and exported to the Statistical Package for Social Science (SPSS) version 25 for analysis. For ease of analysis, the data were coded, recoded, and stored. Descriptive analysis was carried out to describe the characteristics of the respondents based on the study variables. Logistic regression analysis was performed to identify the determinants of neonatal hypoglycemia. The fitness of the regression model was checked using the Hosmer–Lemeshow model fitness test, which indicated a good fit (*p* = 0.129). Multicollinearity among independent variables was checked by the variance inflation factor (VIF) (VIF <10). Variables that were independently associated in bivariable logistic analysis with a *P*-value <0.25 were included in the multivariable logistic regression analysis to control for confounding variables and identify independent determinants of neonatal hypoglycemia. The adjusted odds ratios (AORs) with their respective 95% confidence intervals (CIs) were used, and a *P*-value <0.05 was considered statistically significant. Finally, the results of the study were presented using tables, graphs, and text based on the type of data.

## Results

### Sociodemographic characteristics

A total of 249 participants were included in the study, consisting of 83 cases and 166 controls, resulting in an overall response rate of 97.8%. The mean age of mothers in the case group was 28.94 years, while it was 28.43 years in the control group. Regarding the residence of the study participants, 43.4% of cases were from rural areas, compared to 35.5% of controls. In terms of maternal education, a higher proportion of mothers in the case group (36.1%) had no formal education compared to those in the control group (12.7%). The majority of mothers in the case group were merchants (39.87%), followed by housewives (27.7%). Concerning the timing of neonatal admission, approximately 63.9% of neonates in the case group were admitted between 3 and 5 h of age, with a mean age of 5.04 h; in contrast, only 31.3% of neonates in the control group were admitted within the same timeframe, with a mean age of 6.81 h. Almost all mothers in both the case and control groups had a single pregnancy, and the majority of neonates in both groups were boys, accounting for 53% in the case group and 51.8% in the control group (Table 1).

**Table 1:** Sociodemographic characteristics of study participants at public hospitals in Wolaita Zone, 2023.

Variable	Categories	Cases	Controls	Total
Age of the mother (years)	15–24	18 (21.7%)	29 (17.5%)	47 (18.87%)
	25–34	52 (62.7%)	125 (75.31%)	177 (71%)
	35–49	13 (15.7)	12 (7.2%)	25 (10%)
Residence	Urban	47 (56.6%)	107 (64.5%)	154 (61.84%)
	Rural	36 (43.4%)	59 (35.5%)	95 (38.15%)
Mothers’ occupation	Government employee	10 (12%)	23 (14%)	33 (13.25%)
	Merchant	33 (39.8%)	48 (29%)	81 (32.5%)
	Daily laborer	6 (7.2%)	13 (7.8%)	19 (7.63%)
	Self-employed	11 (13.3%)	20 (12%)	31 (12.44%)
	Housewife	23 (27.7%)	62 (37.3%)	85 (34.13%)
Mothers’ education	No formal education	30 (36.1%)	21 (12.7%)	51 (20.48%)
	Primary education	17 (20.5%)	49 (29.5%)	66 (26.5%)
	Secondary and above	36 (43.4%)	96 (57.8%)	132 (53%)
Sex of the neonate	Male	44 (53%)	86 (51.8%)	130 (52.2%)
	Female	39 (47%)	80 (48.2%)	119 (47.79)
Age of the neonate (h)	3–5	53 (63.9%)	52 (31.3%)	105 (42.16%)
	6–12	30 (36.1%)	114 (68.7%)	144 (57.83%)

### Obstetric characteristics

The study participants in both the case and control groups were multiparous, with 57 participants (68.7%) in the case group and 142 participants (85.5%) in the control group. Nearly all mothers in both groups had at least one antenatal visit, and the majority had four or more visits. Specifically, 51 cases (38.6%) and 142 controls (85.5%) had four or more antenatal visits. Regarding the onset of labor, most mothers in both groups experienced spontaneous labor onset and delivered vaginally in public health institutions. In terms of labor duration, both cases and controls had durations of more than 12 h, with 31% of cases and 12% of controls exceeding this timeframe (Table 2).

**Table 2:** Obstetric characteristics of study participants at public hospitals in Wolaita Zone, 2023.

Variable	Categories	Case	Control	Total
ANC visit	<4	32 (38.6%)	24 (14.5%)	56 (22.48%)
	≥4	51 (38.6%)	142 (85.5%)	193 (77.5%)
Parity	Primipara	26 (31.3%)	24 (14.5%)	50 (20%)
	Multipara	57 (68.7%)	142 (85.5%)	199 (79.91%)
Onset of labor	Spontaneous	74 (89.2%)	154 (92.8%)	228 (91.57%)
	Induced	9 (10.8%)	12 (7.2%)	21 (8.43%)
Mode of delivery	Vaginal delivery	65 (78.3%)	147 (88.6%)	212 (85.14%)
	Cesarean delivery	18 (21.7%)	19 (11.4%)	37 (14.85%)
Place of delivery	Hospital	74 (89.2%)	151 (91%)	225 (90.36%)
	Health center	9 (10.8%)	15 (9%)	24 (9.6%)
Duration of labor (h)	>12	26 (31.3%)	20 (12%)	46 (18.47%)
	<12	57 (68.7%)	146 (88%)	203 (81.52%)

ANC, antenatal care.

### Maternal characteristics

Only 12 mothers (14.5%) in the case group and 5 mothers (3%) in the control group had diabetes. Six mothers (7.2%) in the case group and seven mothers (4.2%) in the control group had pregnancy-induced hypertension (severe preeclampsia). The majority of mothers had normal body mass indices (BMIs), with 67 mothers (80.7%) in the case group and 155 mothers (93.4%) in the control group. Conversely, 11 mothers (6.6%) in the control group and 16 mothers (19.3%) in the case group were classified as overweight.

Among the mothers in the case group, 54 (65.1%) used drugs during pregnancy for various reasons, and 49 (59%) experienced complications such as preterm labor, pre-labor rupture of membranes, oligohydramnios, and others. The most commonly utilized medications were antenatal corticosteroids for preterm labor (Table 3).

**Table 3:** Maternal characteristics of study participants at public hospitals in Wolaita Zone, 2023.

Variables	Categories	Case	Control	Total
Diabetes mellitus	Yes	12 (14.5%)	5 (3%)	17 (6.82%)
	No	71 (85.5)	161 (97%)	232 (93.17%)
Pregnancy-induced hypertension	Yes	6 (7.2%)	7 (4.2%)	13 (5.22%)
	No	77 (92.8%)	159 (95.8%)	236 (94.77%)
Drug use	Yes	54 (65.1%)	46 (27.7%)	100 (40.16%)
	No	29 (34.9%)	120 (72.3%)	149 (59.83%)
Complications during pregnancy	Yes	49 (59%)	39 (23.5%)	88 (35.34%)
	No	34 (41%)	127 (76.5)	161 (64.65%)
BMI (kg/m^2^)	18.5–24.9	67 (80.7%)	155 (93.4%)	222 (89.15%)
	25–29.9	16 (19.3%)	11 (6.6%)	27 (10.84%)

### Neonatal characteristics

Among the admitted neonates, more than half of the cases (55.4%) had a gestational age of less than 36 weeks, while the majority of the controls (77.7%) had a gestational age between 37 and 42 weeks. In terms of birth weight, 43 cases (51%) had a birth weight of less than 2,500 g, while 131 controls (78.9%) had a birth weight greater than 2,500 g at delivery. The majority of the cases (56.6%) were appropriate for gestational age (AGA).

Among the causes for admission, neonatal infection was present in 10 cases (12%), 1 case involved a seizure, 3 cases (3.6%) had congenital defects, and 3 cases (3.6%) had jaundice. None of the cases experienced respiratory distress syndrome, hyaline membrane disease, or meconium aspiration syndrome. Regarding the fifth-minute APGAR score, 41 neonates (49.4%) in the case group scored less than seven, and the majority (84.3%) had axillary body temperatures below 36.5°C. In terms of treatments given to the neonates, 55.4% of cases and 23.5% of controls were administered oxygen, while 12% of cases and 11.4% of controls received antibiotics. In addition, 77.1% of newborns in the case group initiated breastfeeding after 1 h (Table 4).

**Table 4:** Neonatal characteristics of study participants at public hospitals in Wolaita Zone, 2023.

Variables	Categories	Case	Control	Table
Gestational age	Preterm	46 (55.4%)	37 (22.3%)	83 (33.33%)
	Term	37 (44.6%)	129 (77.7%)	166 (66.66%)
Birth weight (g)	<2,500	43 (51%)	35 (21.1%)	78 (31.32%)
	≥2,500	40 (48.2%)	131 (78.9%)	171 (68.67%)
Weight for gestational age	Small for GA	25 (30.1%)	16 (9.6%)	41 (16.46%)
	Large for GA	11 (13.3%)	38 (22.9%)	49 (19.67%)
	Appropriate for GA	47 (56.6%)	112 (67.5%)	159 (63.85%)
Neonatal sepsis	Yes	10 (12%)	13 (7.8%)	23 (9.23%)
	No	73 (88%)	153 (92.2%)	226 (90.76%)
Congenital defect	Yes	3 (3.6%)	3 (1.8%)	6 (2.4%)
	No	80 (96.4%)	163 (98.2)	243 (97.59%)
Jaundice	Yes	3 (3.6%)	5 (3%)	8 (3.21%)
	No	80 (96.4%)	161 (97%)	241 (96.78%)
1st-min APGAR score	<7	5 (6%)	11 (6.6%)	16 (6.42%)
	≥7	78 (94%)	155 (93.4%)	233 (93.57%)
5th-min APGAR score	<7	41 (49.4%)	29 (17.5%)	70 (28.11%)
	≥7	42 (50.6%)	137 (82.5%)	179 (71.88%)
Axillary body temperature	<36.5	70 (84.3%)	61 (36.7%)	131 (52.61%)
	≥36.5	13 (15.7%)	105 (63.3%)	118 (47.38%)
Initiation of breastfeeding	Within 1 h	19 (22.9%)	101 (60.8%)	120 (48.19%)
	After 1 h	64 (77.1%)	65 (39.2%)	129 (51.80%)

### Determinants of neonatal hypoglycemia

All independent variables were analyzed using bivariable logistic regression to assess their association with neonatal hypoglycemia. Variables that showed an association with a *P*-value <0.25 in the bivariable analysis included the following: being an infant of a diabetic mother, age of the neonate at admission, gestational age, birth weight, an axillary body temperature of the neonate, initiation of breastfeeding, 5th-minute APGAR score, mode of delivery, duration of labor, and weight for gestational age.

After controlling for confounders in the final model, the multivariable logistic regression analysis revealed that the following factors were significantly associated with neonatal hypoglycemia in the study area: being a preterm baby [AOR 4.888 (95% CI 1.113–21.478)], age of the neonate at admission between 3 and 5 h [AOR 4.205 (95% CI 1.852–9.547)], hypothermia [AOR 5.485 (95% CI 2.360–12.748)], initiation of breastfeeding [AOR 6.229 (95% CI 2.665–14.599)], mode of delivery [AOR 5.034 (95% CI 1.688–15.011)], and small for gestational age (SGA) [AOR 3.645 (95% CI 1.286–10.330)] (Table 5).

**Table 5:** Multivariable logistic regression analysis indicating determinants of neonatal hypoglycemia among neonates admitted to NICUs of public hospitals in Wolaita Zone, 2023.

Variables	Cases	Controls	COR (95% CI)	AOR (95% CI)	*P*-value
Gestational age					
Preterm	46 (55.4%)	37 (22.3%)	4.335 (2.460–7.638)	4.888 (1.113–21.478)	0.036*
Term	37 (44.6%)	129 (77.7%)	1	1	
Birth weight (g)					
<2,500	43 (51%)	35 (21.1%)	4.024 (2.276–7.112)	1.319 (0.288–6.044)	0.721
≥2,500	40 (48.2%)	131 (78.9%)	1	1	
Weight for gestational age					
Small for GA	25 (30.1%)	16 (9.6%)	3.723 (1.823–7.604)	3.645 (1.286–10.330)	0.015**
Large for GA	11 (13.3%)	38 (22.9%)	0.690 (0.325–1.464)	0.978 (0.334–2.869)	0.968
Appropriate for GA	47 (56.6%)	112 (67.5%)	1		
5th-min APGAR score					
<7	41 (49.4%)	29 (17.5%)	4.612 (2.562–8.302)	2.199 (0.838–5.767)	0.109
≥7	42 (50.6%)	137 (82.5%)	1		
Axillary body temperature					
<36.5	70 (84.3%)	61 (36.7%)	9.269 (4.739–18.127)	5.485 (2.360–12.748)	0.000***
≥36.5	13 (15.7%)	105 (63.3%)	1		
Initiation of breastfeeding					
Within 1 h	19 (22.9%)	101 (60.8%)	1		
After 1 h	64 (77.1%)	65 (39.2%)	5.234 (2.874–9.532)	6.229 (2.665–14.559)	0.000***
Diabetes mellitus					
Yes	12 (14.5%)	5 (3%)	5.442 (1.848–16.026)	4.170 (0.971–17.904)	0.055
No	71 (85.5)	161 (97%)	1		
Mode of delivery					
Vaginal delivery	65 (78.3%)	147 (88.6%)	1		
Cesarean delivery	18 (21.7%)	19 (11.4%)	1.848 (0.895–3.815)	5.034 (1.688–15.011)	0.004**
Duration of labor (h)					
<12 h	26 (31.3%)	20 (12%)	3.330 (1.724–6.432)	1.902 (0.722–5.010)	0.193
>12 h	57 (68.7%)	146 (88%)	1		
Age of the neonate (h)					
3–5	53 (63.9%)	52 (31.3%)	3.873 (2.2236.747)	4.205 (1.852–9.547)	0.001**
6–12	30 (36.1%)	114 (68.7%)	1		

COR: crude odds ratio, AOR: adjusted odds ratio, CI: confidence interval, 1: reference category.

Statistically significant at *P-value <0.05, **P-value <0.01, ***P-value <0.001.

## Discussion

This unmatched case–control study revealed a significant association between hypoglycemia and the following factors: preterm birth, age of the neonate at admission, hypothermia, late initiation of breastfeeding, neonates delivered by cesarean section, and small for gestational age.

In the current study, neonates who were admitted within less than 5 h of their birth were 4.2 times more likely to develop hypoglycemia compared to those admitted between 6 and 12 h. This finding is consistent with research from South Africa and the Netherlands, which reported that most neonates with hypoglycemia experienced it within the first 3 h after delivery and within the first 6 h. This is due to the fact that the transition from intrauterine maternally supported life to extrauterine life accounts for this dip in glucose after birth (20, 21).

According to the current research, neonates with a gestational age of less than 37 weeks were five times more likely to develop hypoglycemia. This finding is in agreement with a study conducted at Hiwot Fana Comprehensive Specialized Hospital, which also found an association between preterm birth and hypoglycemia (15). This finding aligns with multiple studies from Central Java, Nigeria, Israel, Taiwan, Iran, and Iraq. This may be attributed to the fact that preterm newborns may have lower glycogen and fat stores, immature gluconeogenesis pathways, insufficient counter-regulatory responses to hypoglycemia, lower metabolic reserves, and inadequate substrates (9, 13, 18, 19, 22, 23).

Neonates with an axillary body temperature lower than 36.5°C were 5.5 times more likely to suffer from hypoglycemia compared to those with a normal body temperature. This finding aligns with different studies conducted in Ethiopia (Debre Tabor, Harar, and Addis Ababa). The increased risk of hypoglycemia in hypothermic newborns can be attributed to their increased glucose requirements and transition from maternally supported intrauterine life to extrauterine life (8, 15, 19).

The current study observed that neonates who started breastfeeding after an hour were six times more likely to be hypoglycemic compared to those who started within an hour after birth. This finding is consistent with other research findings from Uganda, India, and Ethiopia (11, 15, 24). The possible reason for this association might be that delayed breastfeeding initiation may also result in inadequate milk intake. Breast milk provides essential nutrients, including glucose, to maintain optimal blood sugar levels in newborns. A delay in breastfeeding can lead to missed opportunities for frequent and effective milk intake needed to maintain stable blood sugar levels, increasing the risk of hypoglycemia.

In this study, neonates delivered by cesarean section were five times more likely to suffer from neonatal hypoglycemia. This finding is consistent with findings from Sudan (25), where infants delivered via cesarean section had much lower cord blood glucose levels than those born via vaginal delivery. This result is also in agreement with a report from Iran (26), which reported that hypoglycemia was 2.5 times more prevalent in neonates born via cesarean section compared to those born vaginally. This may be explained by the fact that women may experience fatigue and distress for both themselves and their neonate after a cesarean birth, which can reduce the secretion of breastfeeding hormones.

Being SGA was found to be significantly associated with hypoglycemia in this study. Neonates who were SGA were 4 times more likely to develop hypoglycemia compared to those of appropriate size for gestational age. Similarly, a study from India found an association between hypoglycemia and SGA babies (27). Also, a research study from Pakistan reported a similar result, indicating an association between SGA and neonatal hypoglycemia (28). This might be due to SGA neonates having lower liver glycogen levels, a smaller brain size, poor gluconeogenesis, and hyperinsulinism.

### Limitations of the study

The research utilized existing data from random blood glucose level tests performed on newborns.

## Conclusion

This study identified numerous determinants of neonatal hypoglycemia and provided insights into the most common contributing factors in the study area. The following factors were found to be significantly associated with neonatal hypoglycemia among neonates admitted to NICUs in the study area: prematurity, age of the neonate at admission, axillary body temperature of the neonate less than 36.5°C, initiation of breastfeeding after 1 h, neonates delivered by cesarean section, and small for gestational age.

Therefore, priority should be given to health education on preventive measures, such as early initiation breastfeeding for all newborns, keeping the neonate warm, encouraging immediate skin-to-skin contact, and identifying high-risk groups to lower the risk of hypoglycemia.

To fully understand and prevent the phenomena of hypoglycemia in newborns, additional research with larger sample size and follow-up studies that take into account the limitations of this study are required.

## Data Availability

The raw data supporting the conclusions of this article will be made available by the authors without undue reservation.
